# Self-Reported Versus Observed Measures: Validation of Child Caregiver Food Hygiene Practices in Rural Malawi

**DOI:** 10.3390/ijerph17124498

**Published:** 2020-06-23

**Authors:** Kondwani Chidziwisano, Elizabeth Tilley, Tracy Morse

**Affiliations:** 1 Centre for Water, Sanitation, Health and Appropriate Technology Development (WASHTED), Polytechnic University of Malawi, Private Bag 303, Chichiri, Blantyre 3, Malawi; Elizabeth.Tilley@eawag.ch (E.T.); tracy.thomson@strath.ac.uk (T.M.); 2 Department of Environmental Health, Polytechnic, University of Malawi, Private Bag 303, Chichiri, Blantyre 3, Malawi; 3 Department of Civil and Environmental Engineering, University of Strathclyde, Level 5 James Weir Building, Glasgow G1 1XQ, UK; 4 EAWAG: Swiss Federal Institute of Aquatic Science and Technology, 8600 Dübendorf, Switzerland

**Keywords:** food hygiene, direct observations, self-reported, Malawi

## Abstract

Few studies have attempted to measure the differences between self-reported and observed food hygiene practices in a household setting. We conducted a study to measure the level of agreement between self-reported and observed food hygiene practices among child caregivers with children under the age of five years in rural Malawi. Fifty-eight child caregivers from an intervention and 29 from a control group were recruited into the study. At the end of a nine-month food hygiene intervention, household observations were conducted followed by self-reported surveys. Overall, practices were found to be more frequently reported than observed in both groups. However, the difference between self-reports and observed practices was minimal in the intervention compared to the control group. The odds ratio results confirm that more desirable practices were observed in the intervention group compared to the control group. Despite the effects of reactivity during observations, the study results imply that the intervention group did not just improve their knowledge, but also translated the messaging into better practice. Researchers and implementing agencies in water, sanitation and hygiene and food hygiene sector should ensure that interventions are context-appropriate, and that effective methods of observation are used to confirm any reported effects of an intervention.

## 1. Introduction

Globally, diarrhoeal diseases cause approximately 424,000 childhood deaths annually [1]. Diarrhoeal infections in low and middle income countries (LMIC) have been associated with 9% of childhood mortality annually [2]. Importantly, 62.2% of diarrhoeal deaths in children under the age of five years in LMIC have been associated with poor water quality, sanitation and hygiene, including the consumption of contaminated food at the household level [3,4,5]. Frequent childhood diarrhoea has been associated with stunting, which leads to poor cognitive development in children and reduced economic productivity in adulthood [6,7,8]. In Malawi, high rates of chronic malnutrition have led to 37% of children aged 9–59 months being moderately or severely stunted [9]. In 2016, the Malawi Demographic and Health Survey reported that 22% of children under the age of five had diarrhoea, an increase from the 17.5% reported in 2010 [9,10]. In economically challenged settings, childhood diarrhoea has been linked to various household factors such as faecal contamination of the household environment, animal contact, ingestion of contaminated food and water [11,12,13,14].

Food alone has been suggested to be more important than water in the transmission of diarrhoeal pathogens in some low-income settings [4,15,16,17]. Supplementing breast milk with food, commonly referred to as complementary feeding, at about six months of age [18] has been strongly linked to childhood diarrhoea, due to the foods’ unhygienic preparation [11,19,20,21]. For instance, previous research has reported poor storage of kitchen utensils, storage of left-over food under ambient/high temperature, lack of adequate and/or running water, and contamination of the food preparation areas by domestic animals [11,21,22]. Research in rural Malawi showed that handwashing with soap at critical times, use of clean utensils for serving food and reheating of left-over food are uncommon, and household water and utensils are easily accessed by animals [23,24,25]. Paradoxically, interventions to reduce childhood diarrhoea have tended to focus on water, sanitation and handwashing practices, with little attention on food hygiene and safety at the household level [26]. Child nutrition programmes have also emphasized exclusive breastfeeding and micronutrient supplementation, with little reference to the associated food hygiene practices that should be in place [27].

Despite reports indicating that food hygiene interventions have been effective in reducing childhood diarrhoea, there has been little effort to improve food hygiene practices in rural household settings of LMIC [28,29]. As such, simple, scalable food hygiene behaviour change interventions have been recommended [28,30,31,32,33], which are based and expand on the World Health Organization’s (WHO) five key practices of safer food: keep clean; separate raw and cooked; cook thoroughly; keep food at safe temperatures; and use safe water and raw materials [34].

Childcare in rural household setting in low and middle income countries such as Malawi is mostly done by women (also known as child caregivers) that include mothers, aunts and grandmothers to the children [35,36]. The role of these child caregivers in the health of young children cannot be underestimated since they bear the primary responsibility of cleaning and feeding the children. Thus, it is important that they adhere to the WHO key safer food practices [34] to minimize the ingestion of pathogens associated with food.

In determining the impact and uptake of improved food hygiene practices, one of the key challenges is measuring change in practices, particularly taking into consideration the potential gap between reported and actual practices. As interventions invariably impart knowledge with the aim of changing behaviour and associated practices, the use of knowledge-based reporting assessments, such as questionnaires, can be misleading if interpreted to imply that the gained knowledge has translated into practice. Similarly, as much as structured observations have been found to be effective at recording actual practices [37], they may be affected by the presence of an observer [38]. Although both reported and observation methods have been used to measure food hygiene practices, the level of agreement between these methods in food hygiene studies has not been explored in detail [28,39,40]. Previous research conducted in Burkina Faso, Bangladesh, the Democratic Republic of Congo and the United States of America (USA) showed low levels of agreement between reported and observed hand washing and sanitation practices [41,42,43]. It is important to note that those studies specifically comparing reported and observed food hygiene practices have only been conducted in the USA [44,45], the results of which are not generally applicable to LMICs.

We conducted this study in rural Malawi to measure the level of agreement between the two methods (i.e., observations and interviews) on food hygiene including handwashing practices at the household level. Specifically, we measured the difference between reported and observed practices for both an intervention and control population to validate if the intervention group did not just improve their knowledge, but had also translated the messaging into better practice. This study was undertaken as part of the “Hygienic Family” research project that aimed at improving complementary food hygiene practices in rural households in Malawi [17,46,47].

## 2. Materials and Methods

### 2.1. Study Area

The study was conducted in the rural areas of Chikwawa District, located in the southern region of Malawi. With a population of 564,684 (of which 16% are under the age of 5 years) [48], the district is divided into 12 traditional authorities (TAs), and this study was conducted in three TAs. Two TAs served as the intervention area, while the other one served as a control. Selection of the participating three TAs was completed in collaboration with the Health Department of Chikwawa District Council. Details of the selection criteria have been explained in our previous publications [46].

### 2.2. Study Population and Recruitment

The data used in this paper were collected as part of the end-line survey of a food hygiene intervention [46] that was conducted from November to December 2018 using structured observations and household surveys that included spot checks of water, sanitation and hygiene proxy measures. From the 820 households (Treatment area *n* = 629; Control *n* = 184) who fully participated in the intervention study, including the end-line survey, 58 and 29 households were randomly selected for structured observations in the intervention and control areas, respectively. Self-reported data for this paper were drawn from the household interviews of the same households where the structured observations were conducted. Eligible households had a child under the age of 5 years at the time of data collection, who had participated in the study as either an intervention or control household. The main caregiver of the child was selected as a study participant from each sampled household. The child’s mother (91%) was most often identified as the main child caregiver.

### 2.3. Structured Observations and Household Interviews (Including Household Spot Checks)

At end-line evaluation, the structured observations were conducted within the vicinity of all the sampled 87 households (both in the intervention and control areas). The observations were conducted by six trained female observers (Diploma and BSc holders in Community Development (*n* = 1) or Environmental Health (*n* = 5)); the observers were not involved in the implementation of the intervention and when training them, it was not disclosed as to which one was the intervention and control area. Female observers were chosen because childcare at community level in Malawi is mainly undertaken by women and therefore female observers may gain access to personal information more easily than male ethnographers [49]. To minimize any potential observer effect during observations, the caregivers were told that the purpose of the observations was to learn about childcare without specifying that the focus was on Water, Sanitation and Hygiene (WASH) and food hygiene practices. Observations were conducted from 6 a.m. to 12 p.m. because the formative research findings reveal that the targeted practices were most commonly practiced in the morning hours [24]. With six hours of observation in a small household compound, the observers captured the events of interest.

The practices of interest identified during the formative study were: (1) handwashing with soap at specified critical times (i.e., before child feeding/eating, before food preparation, after cleaning child’s bottom and after latrine use); (2) cleaning utensils with soap; (3) safely storing utensils (i.e., keeping on an elevated place); (4) reheating left-over food; and (5) feeding children by the caregiver [24,25]. The selected practices were previously identified as critical to the improvement of WASH and food hygiene practices at household level [23,30,34,50,51,52]. A structured observation tool [53] guided the development of the pre-coded, structured form that was used to capture all key practices of interest (Supplementary Table S1). In addition, a hand hygiene audit form (Supplementary Table S2) was used to capture handwashing with soap practices. The audit form was structured to capture all handwashing opportunities (including repeated and missed opportunities) performed during the observation period. Each observer conducted about 15 observations in 15 households and they were supervised twice a week by one of the co-principal investigators of the study to ensure consistency in data collection while maintaining data quality.

Two weeks after conducting structured observations, a separate team of enumerators (who were blinded to the treatment allocations) administered a structured questionnaire to the same households to capture information about demographics, child health status, and socio-economic proxy measures. In addition, the questionnaire collected self-reported data from the child caregivers on the same variables (i.e., targeted hygiene practices) that were the focus of the structured observations. At the end of the household interviews, the enumerators conducted spot checks to record hygiene proxy measures such as the presence of a latrine, handwashing facilities (including handwashing facility type, the availability of soap and water), dish racks, domesticated animals, and animal faeces. The interviews were conducted in Chichewa, the local language of the study area. All practice-related questions were in the format of a 5-point Likert scale, since the risk, attitude, norms, ability and self-regulation (RANAS) [54] model of behaviour change was applied in formulating the questions. Example questions are shown in Table 1.

### 2.4. Statistical Analysis

Observational and reported data were cleaned and analysed using Microsoft Excel (Microsoft corporation, Redmond, WA, USA) and Statistical Package for Social Sciences (SPSS), version 25.0 (SPSS Inc., Chicago, IL, USA) respectively. Self-reported practices were compared with directly observed practices by conducting odds ratio and Chi-square tests where the confidence level and probability value (*p* value) were calculated at 95% and <0.05, respectively. The self-reported practice results were divided into the following four categories:

(i) Desirable reported and observed practices: these were desirable self-reported food safety practices confirmed through direct observation;
(ii) Undesirable reported and observed practices: these were undesirable practices observed and then acknowledged through self-report;
(iii) Desirable reported and not observed practices: these were self-reported desirable food safety practices not confirmed through observation;
(iv) Undesirable reported and not observed practices: these were undesirable self-reported food safety practices unconfirmed through direct observation.

For the reported practices measured on the 5-point Likert scale, all responses falling at or below a value of 3 were considered non-performers of the practices and were assigned a “no” response, while those responses at or above 4 were performers of the practices and were assigned “yes” response. Likewise, for the observed practices, participants who were observed as performing the desired practices were assigned a “yes” response, while those who were observed as not performing the desired practices were assigned a “no” response.

### 2.5. Ethical Approval

The research ethics committee of the University of Malawi’s College of Medicine reviewed and approved the study protocol. The study was registered with the Pan African Clinical Trials Registry in March 2017 (PACTR201703002084166). All the study procedures including issues about confidentiality were explained to the caregivers and written informed consent about themselves and that of their children was obtained from them before being included in the study. Upon arrival at the household, the normal rules of the community were followed, where the observer or interviewer greeted members of the household and were offered a place to sit, explained the purpose of the visit, and obtained consent before commencing the observations or interview.

## 3. Results

### 3.1. Demographic Characteristics

Demographic characteristics of the sampled households in both the intervention and control areas were broadly similar for the end line survey (Table 2). The majority of the recruited households in both intervention and control areas had pit latrines. However, more pit latrines and handwashing facilities were found in the intervention area compared to the control group. The study established that some households in the control area did not replace their latrines and handwashing facilities that collapsed during the previous rainy season; however, households in the intervention area continued maintaining their sanitary facilities during and after the rains. It was found that in both groups, households maintained and increased availability of soap for various household uses. Nevertheless, there was no soap available on the handwashing facilities in the control area, while its presence was high in the intervention area (81%, *n* = 47). The presence of animals around households was almost the same in both groups. The mean number of people per household was 5.5 and 5.4 in the intervention and control group, respectively, while the mean age of the child caregivers was 30.8 in the intervention and 28.9 in the control group.

### 3.2. Observed Handwashing Practice 

For handwashing with soap practice, the following pre-specified critical times of handwashing events had been identified as an opportunity to wash hands: before child feeding/eating, before food preparation, after cleaning the child’s bottom, and after latrine use. At the end-line survey, the hand hygiene observations revealed that the number of opportunities (opportunities were considered as all occasions when one was expected to wash hands with soap and water before child feeding/eating; before food preparation; after cleaning child’s bottom; and after latrine use. An opportunity was registered whether soap, water and handwashing facility were available or not) to wash hands at each sampled household was 600 and 313 opportunities in the intervention and control area, respectively, and therefore, proportional to the study population. The results show that there were more opportunities for handwashing ‘before preparing food’ and ‘before child feeding/eating food’ (i.e., main meals and snacks) in both the intervention and control groups (Table 3). However, few opportunities arose to wash hands with soap after cleaning a child’s bottom and after latrine use in both groups, which may have been associated with the time of observation.

### 3.3. Handwashing with Soap Practice at Critical Times

As shown in Table 4, the odds ratio and 95% confidence intervals indicate significant differences in instances of observed handwashing between the intervention and control groups. That is, the study participants in the intervention area were more likely to wash hands with soap during the four critical times of handwashing compared to those in the control area.

A majority (93.1%, *n* = 54) of the households in the intervention area had two handwashing facilities positioned near the latrine (43.9%, *n* = 24) and cooking area (56.1%, *n* = 30), which made it easier for the child caregivers to wash their hands with soap in at critical times. In contrast, one household (3.4%) in the control area had two handwashing facilities.

### 3.4. Frequencies of Observed and Reported Food Hygiene and Handwashing Practices

Table 5 compares the frequencies of reported and observed food hygiene and handwashing practices among the child caregivers in the intervention and control area. In both groups, all practices were found to be more frequently reported than observed. The only exception to this was where children were more frequently observed eating without a spoon than was reported in both groups. The study noted almost similar findings between self-reported and observed practices among the intervention group for handwashing with soap after latrine use, handwashing with soap before food preparation, child feeding/eating with hands, covering of left-over food, keeping utensils in an elevated place, and washing of utensils with soap. These results imply that the practices caregivers reported corresponded with those observed. However, amongst the respondents in the control group, the child caregivers over-reported the practices. The exceptions were for children eating porridge without using a spoon and for the feeding of children by the child caregivers (Table 5).

The study found over-reporting of the following practices in both groups: handwashing with soap after cleaning child’s bottom, handwashing with soap before child feeding/eating, and reheating of left-over food. However, those in the intervention group over-reported these practices more than the control group respondents (Table 5).

### 3.5. Comparison of Observed and Self-Reported Food Hygiene Practices at Individual Level in the Intervention and Control Area

The self-reported desirable food safety practices not confirmed through observation (false positive) were highest amongst the control participants. For instance, 83% (*n* = 24) of the control study participants compared to 19% (*n* = 11) of the intervention participants reported, but were not observed, washing hands with soap before food preparation (Table 6). There were more desirable self-reported food safety practices confirmed through direct observation in the intervention compared to the control group. For instance, 53% (*n* = 31) of the study participants in the intervention area were observed practicing (i.e., washing utensils with soap) what they reported doing. In contrast, 24% (*n* = 7) of participants from the control area were observed practicing what they reported doing (i.e., washing utensils with soap). Furthermore, there were more observed and reported undesirable practices amongst the participants in the control area compared to those in the intervention area. For example, 38% (*n* = 11) of the study participants in the control reported and were observed not washing hands with soap after cleaning a child’s bottom but only 7% (*n* = 4) of subjects from the intervention group were observed and reported not performing the same practice. Importantly, there were some desirable practices which the study participants were observed doing, which they did not self-report during the household interviews. For instance, 22% (*n* = 13) of the study participants in the intervention area were observed feeding their children main meals (i.e., breakfast and lunch). However, these study participants did not report practicing this practice (Table 6). 

## 4. Discussion

This study compared observed and reported findings collected through structured observations and household structured questionnaires regarding practices associated with hand washing, cleaning of utensils with soap, safe storage of utensils (i.e., on an elevated place), reheating of left-over food, and feeding of children by the caregivers in the intervention and control areas. The aim of the study was to the assess validity of data collection methods (i.e., observations and interviews) used in WASH and food hygiene research. With recent trials in WASH being criticized for their findings, which did not establish a relationship between WASH interventions and diarrhoeal reduction/child growth [26,55], ensuring that methods of assessment reflect actual practice is essential. Intervention studies need to be cognizant of using methods that are proven to be effective in promoting children’s health, and this requires an accurate understanding of whether the practices have been put into action.

Similarly to previous work [41,42,43], this study found that participants in the intervention and control groups over-reported targeted practices compared to those observed, demonstrating the effects of social desirability bias. This finding implies that the study participants have hygiene knowledge but tend to report what is desirable, rather than the actual practices they perform. With this in mind, errors associated with over-reporting should be considered when analysing data from self-reported surveys [44]. Similar concerns must also be considered when interpreting observation-based results, as participants may also change their practices if they know that they are being observed. In this study, results about undesirable self-reported food safety practices which were contradicted by the observation of desirable practices on selected practices in both study groups indicate that the study participants changed some of the practices due to the presence of the observer. Conducting observations repeatedly and not revealing the primary purpose of the observation visit have been suggested as possible solutions to address the observer effect [28,41,42]. A study in Burkina Faso confirmed the reliability of repeated observations in addressing the social desirability bias associated with self-reported WASH practices [42].

Our study established that the targeted food hygiene and handwashing practices were more frequently observed and reported by the participants in the intervention group than in the control group. This demonstrates that the intervention not only influenced the level of knowledge, but also the targeted practices among the intervention participants. Similarly, a higher level of food hygiene and safety knowledge was measured in a food safety intervention campaign in Hartford, USA [56]. Nevertheless, the current study established a significant difference between what was reported and observed in both study groups on handwashing with soap before child feeding and reheating of left-over food. The participants knew that it was important to wash hands with soap before feeding their children; however, this was not translated into practice, since most of the targeted children were of an age to self-feed. Under such circumstances, the child caregivers should ensure that their children wash hands with soap before eating. Similarly, despite high knowledge on the importance of reheating left-over food, in reality, challenges in accessing firewood for cooking, and limited time might have contributed to the poor performance of this practice.

In a food safety study conducted in Hartford, USA [44], the agreement of self-reported and observed handwashing practice was low (33%). In our study, the agreement of desirable self-reported food safety practices confirmed through direct observation was higher in all the targeted practices in the intervention group than the control group; there were few undesirable reported and observed practices amongst the intervention participants. A high level of agreement (89%) between self-reported and observed practices was also noted by Kendal et al. [45] in a consumer food behaviour questionnaire validation study. Dharod et al. [56] suggested that the high level of agreement between self-reported and observed practices measured in the study by Kendal et al. [45] could be attributed to the participants’ previous exposure to food hygiene and safety interventions. Similarly, the high level of agreements between self-reported and observed practices in our intervention population may be explained by the fact that participants improved their knowledge and skills through the programme of activities and follow-ups [46]. In particular, the follow-up household visits made by the community volunteers motivated respondents to practice the desired practices consistently to support habit formation. The intervention was designed to be context-appropriate, which may have contributed to the change in the targeted practices in the intervention group. Nevertheless, numerous desirable reported and not observed practices amongst the control participants correspond with previous work [57] indicating that child caregivers have high levels of WASH knowledge, but few are translated into practice.

The type of questions to the respondent has an influence on whether the respondents over-report [42]. Likert scale questions, previously applied in other research [58] to measure psychological constructs, were used in this study to improve the understanding of the questions by the study participants. The Likert scale-based questionnaire provided a range of possible answers (five options) for the study participants to choose their specific responses relevant to the questions rather than if the “yes” or “no” type of responses were used.

Although this study demonstrated over-reporting, the observation of the targeted handwashing and food hygiene practices might have influenced how the study participants behaved due to the presence of the observer [37]. To address this problem, the study participants were told that the purpose of the observations was to learn about daily care of their young children. In addition, the observations were conducted for 6 h per household per day, as an extended duration has been associated with reduced reactivity [59]. In our study, observations were not conducted repeatedly per household due to resource (cost and time) constraints. Similar studies in future should consider conducting the observations repeatedly since this has been proven to strengthen the validity and reliability of observations as a data collection method [42]. Such observation studies could also include more than one observer per session to allow all practices to be fully captured for inter-observer analysis. However, the context in which observations take place should be considered to ensure that conducting observations repeatedly with the presence of additional observers in a small space will not lead to a higher level of social desirability bias. The study population was restricted to 87 households in rural Malawi, which is not statistically representative of rural Malawian households. However, this research provides important information about the validity of the information provided by the study participants, which is necessary to determine the effectiveness of an intervention in changing the targeted practices.

This research has established that the study participants in the intervention area were more likely to wash hands with soap at the targeted critical times of handwashing compared to their counterparts in the control group. Increasing the presence of handwashing facilities in the intervention area was related to the increase in performance of the desired handwashing practice. Encouraging the household participants in the intervention area to install a handwashing facility within the cooking area promoted handwashing before food preparation, an activity rarely performed before the implementation of the intervention [25]. Previous research demonstrated that the presence of handwashing facilities encouraged handwashing practice among community members in Ethiopia [60]. Living in an economically challenged environment might have contributed to the failure of the participating households to practice some of the desired practices (e.g., child eating using spoon). The study established that the majority of the sample population had a low level of education and lived in abject poverty (i.e., below World Bank’s extreme poverty line of USD 1.90 a day [61]), a situation that demands context-specific health promotion approaches to encourage desired WASH and food hygiene practices.

## 5. Conclusions

Our study adds to the evidence that community members have a high level of WASH-related knowledge, but that the knowledge is rarely translated into practice. The development and implementation of this context appropriate intervention for hygiene related practices led to a higher level of agreement self-reported and observed practices within the intervention population. Although there may be still the effect of reactivity during observations, this result implies the intervention group did not just improve their knowledge, but also translated the messaging into better practice. Researchers and implementing agencies in WASH and food hygiene sector should ensure that interventions are context-appropriate, and that effective methods of observation are used to confirm any reported effects of an intervention.

## Figures and Tables

**Table 1:** Likert scale questions for the targeted practices.

Practices	Questions	Answer Format
Hand washing before child feeding/eating	Before you feed your child food (e.g., porridge), how often do you wash your hands with soap and water?	Never Seldom Sometimes Often Very often
Hand washing after using the toilet	After you defecate, how often do you wash your hands with soap and water?	
Hand washing before food preparation	Before you prepare food, how often do you wash your hands with soap and water?	
Hand washing after cleaning child’s bottom	After cleaning child’s bottom, how often do you wash your hands with soap and water?	
Washing kitchen utensils with soap	Before you use kitchen utensils, how often do you wash them with soap and water?	
Keeping kitchen utensils on elevated place	Do you keep your kitchen utensils on an elevated place?	Not at all Somewhat Rather Quite a lot Very much
Reheating of left-over food	Do you reheat left over food before being consumed?	
Feeding of child by the caregiver	Do you feed your child main meals (e.g., lunch and breakfast)?	

**Table 2:** Demographic characteristics of the study population.

Variable	Intervention (N = 58)	Control (N = 29)
Child caregiver is married	88% (*n* = 51)	90% (*n* = 26)
Child caregiver never attended formal education	28% (*n* = 16)	28% (*n* = 8)
Household living on <1.90 $/day	88% (*n* = 51)	83% (*n* = 24)
Presence of animals at household	65% (*n* = 38)	61% (*n* = 18)
Presence of soap at household	91% (*n* = 53)	83% (*n* = 24)
Presence of latrine at household	98% (*n* = 57)	79% (*n* = 23)
Presence of handwashing facility at household	98% (*n* = 57)	14% (*n* = 4)
Soap on handwashing facility	81% (*n* = 47)	0% (*n* = 0)

**Table 3:** Observed (seized and missed) handwashing opportunities.

Critical Time of Handwashing	Opportunities		
	Intervention (600 Opportunities)	Control (313 Opportunities)	X^2^ Test (*p*-Value)
Before child feeding/eating	44.7% (*n* = 268)	54.6% (*n* = 171)	0.391
Before food preparation	42.6% (*n* = 256)	35.8% (*n* = 112)	0.541
After cleaning child’s bottom	3.7% (*n* = 22)	3.2% (*n* = 10)	-
After latrine use	9.0% (*n* = 54)	6.4% (*n* = 20)	-

**Table 4:** Observed handwashing with soap practice at end-line.

Critical Times of Handwashing with Soap	Study Area		Odds Ratio	CI (95%)
	Intervention	Control		
Before child feeding/eating	43.3% (*n* = 116)	0.6% (*n* = 1)	129.7	22.08–5197.5
Before food preparation	47.3% (*n* = 121)	0% (*n* = 13)	6.8	3.57–13.9
After cleaning child’s bottom	72.8% (*n* = 16)	10% (*n* = 1)	24	2.74–558.9
After latrine use	42.6% (*n* = 23)	5% (*n* = 1)	14.1	1.90–610.7

*n* = number (numerator) of occasions the study participants were observed washing hands with soap; the denominator represents the opportunities one was expected to wash hands with soap during the specified critical time of handwashing.

**Table 5:** Observed and reported food hygiene and handwashing practices at end line survey.

Proxy Measures	Control		Intervention	
	Observed	Reported	Observed	Reported
Handwashing with soap after latrine use	5% (*n* = 1)	81% (*n* = 24)	86% (*n* = 50)	96% (*n* = 56)
Handwashing with soap after cleaning child’s bottom	6% (*n* = 2)	69% (*n* = 20)	68% (*n* = 39)	91% (*n* = 53)
Handwashing with soap before food preparation	10% (*n* = 3)	41% (*n* = 12)	68% (*n* = 39)	83% (*n* = 48)
Handwashing with soap before child feeding/eating	3% (*n* = 1)	61% (*n* = 35)	43% (*n* = 25)	95% (*n* = 55)
Child feeding with hands (not using spoon)	39% (*n* = 11)	23% (*n* = 7)	27% (*n* = 16)	19% (*n* = 11)
Child fed by caregiver	35% (*n* = 10)	18% (*n* = 5)	36% (*n* = 21)	18% (*n* = 10)
Left-over food reheated	27% (*n* = 8)	86% (*n* = 25)	49% (*n* = 28)	90% (*n* = 53)
Left-over food covered	52% (*n* = 15)	77% (*n* = 22)	81% (*n* = 47)	93% (*n* = 54)
Keeping utensils on an elevated place	7% (*n* = 2)	29% (*n* = 8)	83% (*n* = 48)	93% (*n* = 54)
Cleaning utensils with soap	24% (*n* = 7)	79% (*n* = 23)	75% (*n* = 44)	88% (*n* = 51)

Intervention group *n*= 58; control group *n* = 29.

**Table 6:** Observed and self-reported food hygiene and handwashing practices at end line.

	Intervention Area (N = 58)				Control Area (N = 29)			
	(Desirable Reported and Observed)	(Undesirable Reported and Observed)	(Desirable Reported and Not Observed)	(Undesirable Reported and Not Observed)	(Desirable Reported and Observed)	(Undesirable Reported and Observed)	(Desirable Reported and Not Observed)	(Undesirable Reported and Not Observed)
Washing utensils with soap	53% (*n* = 31)	0% (*n* = 0)	45% (*n* = 26)	2% (*n* = 1)	24% (*n* = 7)	0% (*n* = 0)	69% (*n* = 20)	7% (*n* = 2)
Keep utensils on an elevated place	76% (*n* = 44)	0% (*n* = 0)	17% (*n* = 10)	7% (*n* = 4)	14% (*n* = 4)	17% (*n* = 5)	52% (*n* = 15)	17% (*n* = 5)
Reheating of left-over food	31% (*n* = 18)	10% (*n* = 6)	59% (*n* = 34)	0% (*n* = 0)	7% (*n* = 2)	10% (*n* = 3)	79% (*n* = 23)	3% (*n* = 1)
Feeding of children by the caregiver	41% (*n* = 24)	14% (*n* = 8)	22% (*n* = 13)	22% (*n* = 13)	14% (*n* = 4)	28% (*n* = 8)	52% (*n* = 15)	7% (*n* = 2)
HW before child feeding/eating	33% (*n* = 19)	0% (*n* = 0)	48% (*n* = 28)	19% (*n* = 11)	0% (*n* = 0)	28% (*n* = 8)	62% (*n* = 18)	10% (*n* = 3)
HW Before food preparation	64% (*n* = 37)	2% (*n* = 1)	19% (*n* = 11)	16% (*n* = 9)	0% (*n* = 0)	17% (*n* = 5)	83% (*n* = 24)	0% (*n* = 0)
HW After cleaning child’s bottom	53% (*n* = 31)	7% (*n* = 4)	38% (*n* = 22)	2% (*n* = 1)	3% (*n* = 1)	38% (*n* = 11)	59% (*n* = 17)	0% (*n* = 0)
HW After latrine use	53% (*n* = 31)	3% (*n* = 2)	43% (*n* = 25)	0% (*n* = 0)	3% (*n* = 1)	41% (*n* = 12)	55% (*n* = 16)	0% (*n* = 0)

Desirable reported and observed practices: these were desirable self-reported food safety practices confirmed through direct observation; Undesirable reported and observed practices: these were undesirable practices observed and then acknowledged through self-report; Desirable reported and not observed practices: these were self-reported desirable food safety practices not confirmed through observation; Undesirable reported and not observed practices: these were undesirable self-reported food safety practices unconfirmed through direct observation.

## Supplementary Materials

The following are available online at https://www.mdpi.com/1660-4601/17/12/4498/s1, Table S1: Household structured observation form, Table S2: Hand washing audit form.
