# Supplementary material for: Self-Reported Versus Observed Measures: Validation of Child Caregiver Food Hygiene Practices in Rural Malawi
Source: Int J Environ Res Public Health. 2020 Jun 23;17(12):4498. doi: 10.3390/ijerph17124498 (PMC7344643; doi:10.3390/ijerph17124498)
Supplement: Supplementary file 1 [file ijerph-17-04498-s001.pdf]

Structured Observation Checklist (Household)

Informed Consent Form

Good morning! My name is ______________________. I am working for ……, a non-profit, non-governmental organization, based in …… Currently, we are conducting the study on **"………."** in x districts of ……. (……..) for WASHTED and SHARE.

The main objective of this study is to learn on how you care for child during the day. In order to do so, we would like to observe your daily activities for about 6 hours. We will observe in those houses of mother (or primary care takers of children if mother is not available) who have child aged 18-59 months. Your participation is voluntary. You can stop at any point during the observation.

We would be grateful if you allow us for the observation since this observation will be extremely important and will contribute to our study. During the observation you do not need to do any additional thing just continue your normal/routine day practices/work.

Do you have any questions? Yes No

Do I have your consent to begin the observation now? Yes No

Observers visits record			
Date of interview			
Household ID			
Name of observer	…………………………	………………………………	……………………………...
Observation result<br><br>*(Instruction; please mention reason behind refusing)*	Observation completed…………………………......1<br>Not met concerned person for observation……….2<br>Said to observe next time……………………...........3<br>Nobody met at home ……..…….……….................4<br>Observation Incomplete……………………….........5<br>Refused for observation.......................................6<br>Other (specify)..		

Note for observer: *Please make the purpose of your visit anonymous. Don't use structured observation checklist in-front of mother. Only use notebook to make notes and record observed behaviours during structured observation. Don't give any signal/clue that you are observing mother's hygiene key behaviours. You will only mention your purpose of visit is to know the 'daily routine of mothers and to know the diarrhoea status of their children aged between 18-59 months in last two week'. Make sure that you are observing mother's hygiene behaviour in-between 6-12 noon and mark in the checklist accordingly. Only observe one household per day.*

Structured observation starts at 6am or 1pm:
Note for observer: *don't ask any question to mother. Only observe mother or primary care takers of children if mother is not available) behaviours but not the behaviours of other family members in house. If mother (or primary care taker) is not available at home, go to next participating household. If mother (or primary care taker) is available at home, briefly talk about her daily routine or any other local functions/rituals but don't talk too much. Observe behaviours as reflected in the structured observation checklist below. Only circle the correct answer soonest after you complete the observation in respective house.*

Section 1: Household/Cluster Identification

Q. N	Question	Response	Code	Instruction
1	District name	☐☐ ___________		
	TA name	☐☐		
104	Village name			

Observe the behavioural practices of mother or primary caretakers of children having children from 18-59 months

Now I am going to start my observational works.

Water storage and treatment practice				
**Q.N**	**Questions**	**Response**	**Code**	**Instruction**
1.	What is the main source of drinking water used by the household?	Piped water in residence	1	
		Piped water to tap in yard, plot	2	
		Borehole	3	
		Protected spring	4	
		Surface water	5	
		Water vendor	6	
		Other (specify)……………………………..		
2.	What is the main source of water for domestic purposes (e.g. washing utensils) used by the household?	Piped water in residence	1	
		Piped water to tap in yard, plot	2	
		Borehole	3	
		Protected spring	4	
		Surface water	5	
		Water vendor	6	
		Other (specify)……………………………..		
3.	How is drinking water stored within the household?	Covered Barrel	1	
		Open barrel	2	
		Covered bucket	3	
		Open Bucket	4	
		Covered Mtsuko	5	
		Open mtsuko	6	
		Other (Specify)…………………………..	99	
4.	How is water for other domestic purposes (e.g. for washing utensils) stored within the household?	Covered Barrel	1	
		Open barrel	2	
		Covered bucket	3	
		Open Bucket	4	
		Covered Mtsuko	5	
		Open mtsuko	6	
		Other (Specify)…………………………..	99	

5.	What is the condition of the stored water?<br>(*Clean means absence of clay, mud, turbidity in water, things like dust, animal hair, insects and any visible residue, color objects in or on the surface of the water)*	Visibly Clean	1	
		Visibly Dirty	0	
6.	What is used to wash the storage container	Water only	1	
		Water and soap	2	
		Water and mud	3	
		Water and ash	4	
		Others (specify)	99	
7.	What is used to wash the water collection container	Water only	1	
		Water and soap	2	
		Water and mud	3	
		Water and ash	4	
		Others (specify)	99	
8.	Where is drinking water storage container placed within the household	Inside the house on a raised place	1	
		Inside the house on the floor	2	
		Outside the house on a raised place	3	
		Outside the house on the ground	4	
		Others (specify)	99	
9.	Where is water storage container with water for domestic purposes (e.g. water for cleaning utensils) placed within the household	Inside the house on a raised place	1	
		Inside the house on the floor	2	
		Outside the house on a raised place	3	
		Outside the house on the ground	4	
		Others (specify)	99	
10.	Are drinking water containers/pots properly covered with covering lid?<br>*(Properly covered means complete covering of the container)*	Yes	1	
		No	0	
11.	Can drinking water be accessed by animals	Yes	1	
		No	0	
12.	Was drinking water accessed by animals during observation period	Yes	1	
		No	0	

13.	Can water for other domestic purposes (e.g. washing utensils) be accessed by animals	Yes	1	
		No	0	
14.	Was water for other domestic purposes (e.g. washing utensils) accessed by animals during observation	Yes	1	
		No	0	
15.	Which animals accessed the water	Poultry	1	
		Pig	2	
		Goat	3	
		Cow	4	
		Dog	5	
		Cat	6	
		Others specify	99	
16.	How do they get the drinking water (e.g. using two cup system, one cup)<br>Explain in detail			
**Sanitation and faeces management**				
17.	Does the household have a toilet?	Yes	1	
		No	0	
18.	Does the toilet have a drop hole cover	Yes	1	
		No	0	

19.	Assess condition of toilet by observing the following:<br>*(Instruction: "Clean" means that the floor, drop hole and walls of the toilet are visibly clean)*	Yes	No		
		1	0		
A	*Floor of toilet*	1	0		
B	*Drop hole surrounding*	1	0		
C	*Wall of toilet*	1	0		
D	*Flies seen*	1	0		
E	*Bad smelling*	1	0		

20.	Do all the family members use the toilet while they are at house?<br>*(Instruction: if all members use toilet put 'always', if few and few*	Always	1	
		Sometimes	2	
		Never	3	

	not 'sometimes' and 'never' – if no one used it)			
21.	Is human faeces' observed in the household premises?	Yes	1	
		No	0	
22.	Where child aged 18 - 59 months defecate?	In open place (anywhere)	1	
		Potties	2	
		Nappies/diapers	3	
		Clothes	4	
		Others (Specify)…………………..		
23.	How the child's faeces disposed?	Picked up and disposed in toilet	1	
		Picked up and disposed in dumping side	2	
		Left as it is	3	
		Covered with soil	4	
		Throw anywhere	5	
		Wash in bucket	6	
		Others (specify).....................................		
24.	If disposed in toilet, what is used to carry the faeces	Hoe	1	
		Leaves	2	
		Piece of metal	3	
		Piece of paper	4	
		Bare hands	5	
		Others (specify)	99	
25.	If faeces were removed from where the child defecated and disposed somewhere, are some faeces still remain on the disposal place?	yes	1	
		No	0	
26.	How are soiled nappies/clothes treated	Washed immediately when soiled	1	
		Packed in bucket to be washed later	2	
		Packed on another place e.g. roof to be washed later	3	
		Others specify	99	
Hand Washing				
27.	Is there a specific hand washing station/area?	Yes	1	
		No	0	

28.	What is the location of the handwashing facility	Inside or near the latrine	1	
		Under the dish rack	2	
		near the cooking place/fire	3	
		Elsewhere inside the house	4	
		Outside the house near the door	5	
		Elsewhere in the compound	6	
		Others (Specify)	99	
29.	Are soap and water available in the hand washing station/area?	Only Soap	1	
		Only Water	2	
		Both Soap and Water available	3	
		Both Soap and Water unavailable	4	
		Ash and water	5	
		Other(Specify)…………………………		
30.	What type of soap is it?	Liquid soap	1	
		Bar soap	2	
		Powdered soap	3	
		Ash	4	
		Flour	5	
		Others (Specify)	99	
31.	Is anyone at the household wearing a handwashing with soap bracelet?	Yes	1	
		No	0	
32.	If yes, who is wearing the bracelet	Mother	1	
		Father	2	
		Grandmother	3	
		Grandfather	4	
		Sibling	5	
		Others (Specify)	99	

**Food Storage and Food Hygiene**				
33.	Where is food mostly prepared at the household	Kitchen	1	
		Veranda	2	
		Household yard	3	
		Inside the house	4	
		Others (specify)	99	
34.	Observe steps followed when preparing porridge for the child *(insert the number inorder of responses)*		No.	How many times

	Kodi mungandiuze ndondomeko yomwe mumatsatira mukamakonza chakudya chanu (kuyambira poyamba mpaka pamapeto)	Wash pot and other utensils with soap		
		Wash pot and other utensils without soap		
		Put washed utensils on raised place		
		Put washed utensils on lower place		
		Set up fire		
		Caregiver wash hands with soap		
		Wash hands without soap		
		Add water in pot and put it on fire		
		Cooking porridge: add flour & stir		
		Remove pot from fire		
		Pot with porridge covered		
		Serve porridge in clean washed utensils		
		Serve porridge in dirty plate		

		Caregiver washed hands with soap		
		Child washed hands with soap		
		Caregiver washed hands without soap		
		Child washed hands without soap		
		Feed the child porridge with spoon		
		Feed the child with hand		
		Child self-feeding with spoon		
		Child self – feeding with hands		
35.	Who prepares child food	Mother	1	
		Father	2	
		Grand mother	3	
		Grand father	4	
		Sibling	5	
		Others	99	
36.	Who feeds the child porridge	Mother	1	
		Father	2	
		Grand mother	3	
		Grand father	4	
		Sibling	5	

		Self-feeding	6	
		Others	99	
37.	Who feeds the child lunch (e.g. nsima)	Mother	1	
		Father	2	
		Grand mother	3	
		Grand father	4	
		Sibling	5	
		Self-feeding	6	
		Others	99	
		Not observed	101	
38.	By any chance, have you observed a baby bib being used during child feeding?	Yes	1	
		No	0	
39.	What lunch was served			
40.	Was lunch freshly prepared or stored (i.e. both relish and nsima/rice)			
41.	Who feeds the child snacks and fruits	Mother	1	
		Father	2	
		Grand mother	3	
		Grand father	4	
		Sibling	5	
		Self-feeding	6	
		Others	99	
		Not observed	101	
42.	What type of snacks were given to the child			
43.	Were the snacks clean? E.g. washed			
44.	What is used when child is eating porridge	Spoon	1	
		Hand	2	
		cup	3	
		Plate	4	
		Both spoon and hands	99	
45.	Is any cooked leftover/stored food at home/Kitchen observed?	Yes	1	

	(Note: Make sure that you will observe all the potential areas of food storage.)	No	0	
46.	What type of left-over food has been stored	Nsima	1	
		Porridge	2	
		Relish	3	
		Potatoes	4	
		Cassava	5	
		Sorghum	6	
		millet	7	
		Rice	8	
		Others (specify)	99	
47.	Is leftover/stored food/cooked food kept covered with covering lid?<br>*(Note: If all foods were covered then only mark as Yes. If some covered and some not then mark No)*	Yes	1	
		No	0	
		Some	2	
48.	Is the covering lid of leftover/ stored/ cooked food clean?<br>(*Clean means free from dirt, dust, flies and insects)*	Yes	1	
		No	0	
49.	Is leftover food re-heated before feeding to child?	Yes	1	
		No	0	
		Not observed	101	
50.	Is the stored food thoroughly reheated before feeding?<br>*(Note: Thoroughly re-heat means, observation of time taken to re-heat. Time might vary by types of food, volume of food and intensity of fire. Liquid food should be rolling boiled and in case of solid food there should be visible steam coming from food. If food is re-heated in bowl or plate, circle 'no')*	Yes	1	
		No	0	
		Not observed	101	
51.	Are serving utensils (*plate, bowl, glass and spoon*) clean before serving food?<br>*(Instruction: This evidence can be seen multiple times. If mother missed at any times, please write 'no')*	Yes	1	
		No	0	
		sometimes	2	
52.	If yes, what was used to wash the utensils? *(Observe at all times when the utensils are being washed)*			

53.	Are cooked and uncooked foods kept separately?	Yes	1	
		No	0	
54.	Where are the utensils kept when inside the house	On the floor	1	
		In basin on the floor	2	
		On top of a bag of maize, millet	3	
		In basin on a chair	4	
		On a modern dish rack	5	
		In basin on a crate	6	
		In cup board	7	
		Others (specify):	99	
55.	Can the utensils accessed by animals on this place	Yes	1	
		No	0	
56.	Is there a dish rack at the household	Yes	1	
		No	0	
57.	Where are the utensils kept when outside the house	On the ground	1	
		In basin on the ground	2	
		In basin on a chair	3	
		On a modern dish rack	4	
		On Thandala	5	
		In basin on a crate	6	
		Others (specify)	99	
58.	Can domestic animals access utensils from this place	Yes	1	
		No	0	
59.	Are there domestic animals at the household	Yes	1	

		No	0	
60.	What domestic animals are present	Poultry	1	
		Pig	2	
		Cattle	3	
		Goat	4	
		Sheep	5	
		Dog	6	
		Cat	7	
		Others	99	
61.	Are animal faeces present inside the house	Yes	1	
		No	0	
62.	Are animal faeces present outside the house	Yes	1	
		No	0	
63.	The droppings are from which animals?			
64.	Observe if the household has a raised place for storage of utensils inside the house?	Yes	1	
		No	0	
65.	Have you observed if the food hygiene buntings are hanged anywhere?	Yes	1	
		No	0	
66.	If yes where exactly are they hanged?	Window	1	
		Door	2	
		Sitting room	3	
		Kitchen	4	
		Veranda	5	
		Others (Specify)	99	
67.	By any chance have you seen a Banja la ukhondo badge?	Yes	1	
		No	0	

Please write down your observation note based on your observation (example: nappy not immediately washed after changed, mother might re-heat few foods, few not, mother might cleans few serving utensils few not, mother might have multiple exposure during feeding etc):

.

Thank you so much for your valuable time

share
Sanitation and Hygiene Applied Research for Equity

UNIVERSITY OF MALAWI

washted
Centre for Water, Sanitation, Health & Appropriate Technology Development

Hand Hygiene Observation Form

Date : **HH ID :** **District :**

TA : **Village :** **Observer Name :**

**Op.**	**Person**	**Event**	**HH Action**	**Handwashing facility**	**HWF location**	**Comments**
	☐ Mother<br>☐ Sibling<br>☐ Father<br>☐ G/parent<br>☐ Child<br>☐ Other Specify:	☐ child feeding<br>☐ before eating<br>☐ Food preparation<br>☐ changing nappy<br>☐ after latrine use<br>☐ Touch dirty things: Specify:<br><br>☐ Other Specify:	☐ water only<br>☐ water with soap<br>☐ water with ash<br>☐ water with sand<br>☐ water with flour<br>☐ No action	☐ Tippy tap<br>☐ Jug and basin<br>☐ bucket without tap<br>☐ bucket with tap<br>☐ cup only<br>☐ Deep in basin/bucket<br>☐ Other Specify:	☐ outside toilet<br>☐ Under dish rack<br>☐ in HH yard<br>☐ Inside the house<br>☐ Other Specify:	
**Op.**	**Person**	**Event**	**HH Action**	**Handwashing facility**	**HWF location**	**Comments**
	☐ Mother<br>☐ Sibling<br>☐ Father<br>☐ G/parent<br>☐ Child<br>☐ Other Specify:	☐ child feeding<br>☐ before eating<br>☐ Food preparation<br>☐ changing nappy<br>☐ after latrine use<br>☐ Touch dirty things: Specify:<br><br>☐ Other Specify:	☐ water only<br>☐ water with soap<br>☐ water with ash<br>☐ water with sand<br>☐ water with flour<br>☐ No action	☐ Tippy tap<br>☐ Jug and basin<br>☐ bucket without tap<br>☐ bucket with tap<br>☐ cup only<br>☐ Deep in basin/bucket<br>☐ Other Specify:	☐ outside toilet<br>☐ Under dish rack<br>☐ in HH yard<br>☐ Inside the house<br>☐ Other Specify:	
**Op.**	**Person**	**Event**	**HH Action**	**Handwashing facility**	**HWF location**	**Comments**

	☐ Mother<br>☐ Sibling<br>☐ Father<br>☐ G/parent<br>☐ Child<br>☐ Other Specify:	☐ child feeding<br>☐ before eating<br>☐ Food preparation<br>☐ changing nappy<br>☐ after latrine use<br>☐ Touch dirty things: Specify:<br><br>☐ Other Specify:	☐ water only<br>☐ water with soap<br>☐ water with ash<br>☐ water with sand<br>☐ water with flour<br>☐ No action	☐ Tippy tap<br>☐ Jug and basin<br>☐ bucket without tap<br>☐ bucket with tap<br>☐ cup only<br>☐ Deep in basin/bucket<br>☐ Other Specify:	☐ outside toilet<br>☐ Under dish rack<br>☐ in HH yard<br>☐ Inside the house<br>☐ Other Specify:	
**Op.**	**Person**	**Event**	**HH Action**	**Handwashing facility**	**HWF location**	**Comments**
	☐ Mother<br>☐ Sibling<br>☐ Father<br>☐ G/parent<br>☐ Child<br>☐ Other Specify:	☐ child feeding<br>☐ before eating<br>☐ Food preparation<br>☐ changing nappy<br>☐ after latrine use<br>☐ Touch dirty things: Specify:<br><br>☐ Other Specify:	☐ water only<br>☐ water with soap<br>☐ water with ash<br>☐ water with sand<br>☐ water with flour<br>☐ No action	☐ Tippy tap<br>☐ Jug and basin<br>☐ bucket without tap<br>☐ bucket with tap<br>☐ cup only<br>☐ Deep in basin/bucket<br>☐ Other Specify:	☐ outside toilet<br>☐ Under dish rack<br>☐ in HH yard<br>☐ Inside the house<br>☐ Other Specify:	
